# Medical Officers Object to Whole-Time Service

**Published:** 1920-08-28

**Authors:** 


### Medical Officers Object to Whole-time Service.

Several Boards of Guardians have adopted new arrangements regarding the medical treatment of the sick poor who reside at their own homes. In place of the district medical officers, who are part-time officials, being also in private practice, the medical treatment of the outdoor sick poor has been placed in the hands of whole-time resident medical officers on the staff at the various infirmaries.

The Poor-Law Medical Officers' Association has now passed a resolution deploring the attempt to eliminate part-time district medical officers, which, it is claimed, would be of disadvantage to the sick poor.

This resolution has been sent to all Boards of Guardians, and the suggestion is made that no changes of this kind should be made until the whole subject has been carefully considered and the best scheme for the general welfare decided on. If the scheme recommended by Lord Dawson's Committee becomes an established fact, it is urged that the need for the Poor-Law system of medical treatment for the outdoor sick poor will not exist, as all citizens would then have the right to medical treatment.

